# Utilization of FAD-Glucose Dehydrogenase from *T. emersonii* for Amperometric Biosensing and Biofuel Cell Devices

**DOI:** 10.1021/acs.analchem.1c02157

**Published:** 2021-08-12

**Authors:** Roy Cohen, Rachel E. Bitton, Nidaa S. Herzallh, Yifat Cohen, Omer Yehezkeli

**Affiliations:** † Faculty of Biotechnology and Food Engineering, Technion—Israel Institute of Technology, Haifa 3200003, Israel; ‡ Russell Berrie Nanotechnology Institute, Technion—Israel Institute of Technology, Haifa 3200003, Israel; § The Nancy and Stephen Grand Technion Energy Program, Technion—Israel Institute of Technology, Haifa 3200003, Israel

## Abstract

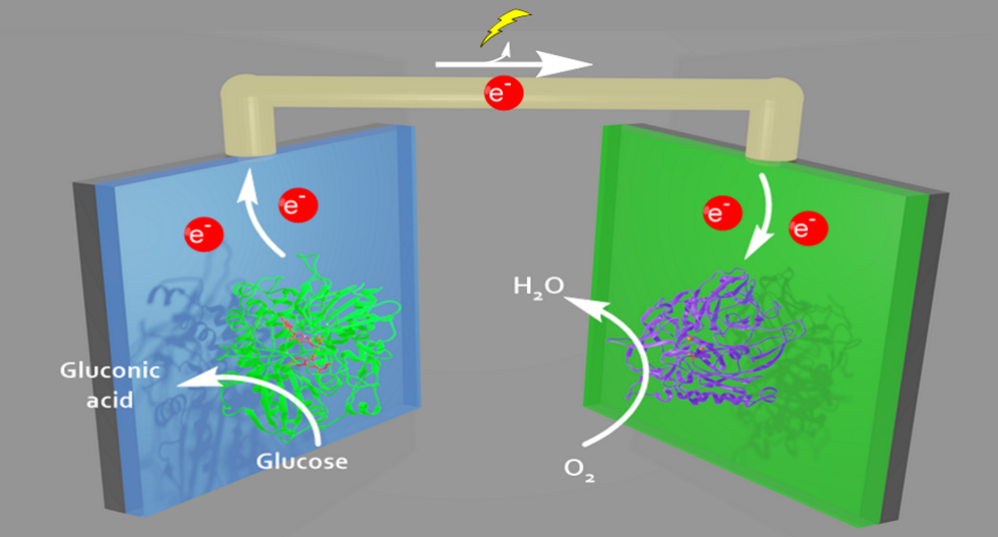

Flavin-dependent glucose dehydrogenases (FAD-GDH) are oxygen-independent enzymes with high potential to be used as biocatalysts in glucose biosensing applications. Here, we present the construction of an amperometric biosensor and a biofuel cell device, which are based on a thermophilic variant of the enzyme originated from *Talaromyces emersonii*. The enzyme overexpression in *Escherichia coli* and its isolation and performance in terms of maximal bioelectrocatalytic currents were evaluated. We examined the biosensor’s bioelectrocatalytic activity in 2,6-dichlorophenolindophenol-, thionine-, and dichloro-naphthoquinone-mediated electron transfer configurations or in a direct electron transfer one. We showed a negligible interference effect and good stability for at least 20 h for the dichloro-naphthoquinone configuration. The constructed biosensor was also tested in interstitial fluid-like solutions to show high bioelectrocatalytic current responses. The bioanode was coupled with a bilirubin oxidase-based biocathode to generate 270 μW/cm^2^ in a biofuel cell device.

Diabetes has become a major threat to public health worldwide, and therefore, methods to minimize its effects on the population’s life span and life quality should be developed. As a prerequisite, tight regulation of the patient’s glucose levels is required. Glucose bioelectrochemical sensing has been studied extensively for the last several decades.^1−3^ Glucose oxidase (GOx) has been the most commonly used biocatalyst for direct or indirect analyte monitoring. GOx oxidizes glucose while reducing O_2_ into H_2_O_2_, which is subsequently reduced or oxidized on an electrode.^4−6^ As the oxidation of one glucose correlates to one hydrogen peroxide molecule, the concentration of glucose is inferred from the bioelectrocatalytic current caused by the H_2_O_2_ electrochemical reaction. Though this method was successfully implemented in biomedical devices, GOx still required oxygen for its activity, a gas whose concentration changes over time. To address this issue, several methodologies were developed, e.g., addition of a redox mediator or a polymeric chain with redox-active moieties that facilitate the electron transfer (ET) process.^7−11^ However, dissolved oxygen can still interfere with the measurements and reduce the biosensor’s accuracy. As an alternative, dehydrogenase enzymes were also tested as oxygen-insensitive glucose oxidation catalysts. Enzymes such as nicotinamide adenine dinucleotide (NAD) and pyrroloquinolinequinone (PQQ)-dependent glucose dehydrogenase (GDH) were oxygen-independent and robust, yet problematic. NAD-GDH has a diffusible cofactor that complicates device fabrication, and PQQ-GDH has low substrate specificity and calcium is required for its activation.^12−16^

In contrast, flavin adenine dinucleotide-dependent GDH (FAD-GDH) is oxygen-independent, specific, and is not impaired by the issues mentioned above. Thus, FAD-GDH is an attractive biocatalyst for amperometric biosensing and biofuel cell (BFC) devices. In the last several decades, FAD-GDH was isolated from different origins and further characterized. Just in the last decade, several integrated configurations have been shown.^17−21^ These systems were fabricated using electropolymerization of aniline^22^ or entrapment techniques that include a polymeric backbone with an Os complex or quinones as redox mediators.^23,24^ The developed systems enabled an efficient mediated electron transfer (MET) process between the FAD-GDH enzyme and the electrodes. FAD-GDH was also bioengineered to fit direct electron transfer (DET) processes. Alfonta and co-workers fused minimal cytochrome *c* to the FAD-GDH α subunit.^25^ Sode and co-workers chose a similar approach to develop a DET glucose biosensing device using a GDH variant from *A. flavus*.^26^ In both cases, the linear range was limited and the bioelectrocatalytic currents were lower as compared to MET systems. Both systems also required high overpotential for activation. For glucose biosensing and BFC devices, minimal overpotential is a crucial parameter for false-positive sensing or gaining maximal power outputs, respectively.^27^ Recently, the overexpression of FAD-GDH from *Talaromyces emersonii* (TeGDH) in *Escherichia coli* was presented.^28^ The enzyme is structurally similar to another GDH variant from *A. flavus*(29) and has ideal properties for glucose biosensing, mainly suitable pI, optimal pH, specificity, and *K*_M_ values. While some advances have been presented toward the development of amperometric glucose sensing using TeGDH,^30^ methods to structurally support the enzyme on the electrodes and its activation at low overpotential are required to allow continuous operation. Presented here are the fabrication and development of an amperometric glucose biosensor based on TeGDH. The full process from enzyme cloning to sensor fabrication is detailed.

Furthermore, the developed biosensor was further coupled to a bilirubin oxidase-based biocathode and utilized in a biofuel cell configuration. The developed sensor has shown oxygen-independent activities with a linear response to glucose concentrations in the required 0–20 mM range. Various interferents were also tested, showing minimal effects on sensing. Moreover, the developed sensor shows at least 20 h of a continuous operational lifetime with a low decrease in current. An enzymatic BFC (EBFC) was constructed leading to a production of power outputs of 270 μW/cm^2^ in the presence of enriched O_2_ and 63 μW/cm^2^ without. Both bioanode and biocathode used polydopamine (PDA) and redox mediators as a cost-effective matrix for enzyme encapsulation and ET, respectively.

## Experimental Section

For a more detailed description, please see the Supporting Information (SI). The SI contains the characterization of TeGDH, including plasmid sequence, absorbance, sodium dodecyl sulfate-polyacrylamide gel electrophoresis (SDS-PAGE), and Michaelis–Menten curve. Additionally, the TeGDH bioanode is further characterized by quantification of enzyme and mediator adsorption. Construction of the TeGDH bioanode using other redox mediators and surfaces is also detailed.

### Chemicals and Instrumentation

Glassy carbon electrodes (GCE, 3 mm diameter) were purchased from CH Instruments. Dichlorophenolindophenol (DCPIP), dopamine, uric acid, thionine acetate, and d-glucose were purchased from Sigma-Aldrich. Dimethylformamide (DMF) was purchased from Bio-Lab. Multiwalled carbon nanotubes (MWCNTs) were purchased from NanoIntegris (MWCNTs, 99 wt %, <20 nm. OD). 2,3-Dichloro-naphthoquinone (DCNQ) 98% was purchased from Acros Organics. Acetaminophen was obtained via crushing a 500 mg commercial paracetamol tablet (Teva Pharmaceuticals, Israel). Bilirubin oxidase (BOD) from *Myrothecium verrucaria*, and 2,2′-azino-bis(3-ethylbenzothiazoline-6-sulfonic acid) (ABTS) were purchased from SIGMA Life Science. Ascorbic acid was purchased from Thermo Fischer. All chemicals and reagents were used without further purification.

All graphs were prepared using Origin software (OriginLab). Electrochemical measurements were executed with a BioLogic SP-200 potentiostat, supported by EC-Lab software (BioLogic, France). Protein purification was performed using AKTA GO FPLC (Cytiva) equipped with a Superdex 200 column (Cytiva).

### Enzyme Production

The thermophilic *T. emersonii* glucose dehydrogenase (TeGDH) gene was externally synthesized and cloned into pET29b (Twist Bioscience) using the sequence from entry LC069047 (DDBJ) after codon optimization and signal peptide removal (AA 2–17). Competent *E. coli* BL-21 (DE3) cells were transformed with pET29TeGDH and negatively selected using kanamycin agar plates. Surviving colonies were positively selected via a colony polymerase chain reaction (PCR), using the following primers: forward—5′ TTA TGC GAC TCC TGC ATT AG 3′ and reverse—5′ GTG CCA TAT GTA TAT CTC CTT C 3′. For TeGDH overexpression, 50 mL of LB starter of BL21DE3/pET29TeGDH was inoculated with 50 μg/mL kanamycin and then incubated at 37 °C, 180 rpm, overnight. The entire starter was mixed into a 1 L Erlenmeyer flask containing 500 mL of terrific broth (without glycerol), which was inoculated with 50 μg/mL kanamycin and then incubated at 37 °C, 180 rpm, overnight. The entire starter was mixed into a 1 L Erlenmeyer flask containing 500 mL of terrific broth (without glycerol), which was inoculated with 50 μg/mL kanamycin, 1 mM MgCl, 1 mM CaCl, and 10 mM glucose. The cells were further incubated at 37 °C, 180 rpm for approximately 7 h. Then, the cells were induced with 500 μL of 0.3 M isopropyl β-d-1-thiogalactopyranoside (IPTG) for 18–20 h of incubation while stirred at 25 °C. The cells were centrifuged and the pellet was kept at −80 °C.

TeGDH cells from a frozen tube were resuspended in 20 mL of lysis buffer (50 mM KPi pH 7.5, 10 mM imidazole, and 300 mM NaCl) and disrupted by ultrasonication (30% amplitude, 15 s on 30 s off, 11 min total on). Removal of cell debris was performed by centrifugation (10 000*g*, 30 min, 4 °C), after which the supernatant was kept. The supernatant was passed through a Ni-NTA column, equilibrated with 20 mM KPi pH 7.5 containing 25 mM imidazole and 300 mM NaCl. The enzyme was eluted with 200 μL aliquots of 20 mM KPi pH 7.5, 300 mM imidazole, and 300 mM NaCl. The two main fractions were combined and further purified using a Superdex 200 column, equilibrated with 20 mM KPi pH 7.5 containing 150 mM NaCl. Using an isocratic gradient at 0.5 mL/min, the protein was eluted after ∼16 mL. The main fractions of TeGDH were combined and concentrated using a PALL centricon (4000 rpm, 4 °C, 30 min, 10 kDa cutoff).

### Biosensor Construction and Measurement

GCEs were polished with 1 and 0.05 μm of alumina beads in a sequence. A suspension of MWCNTs (5 mg/mL) was prepared by dissolving MWCNTs in DMF, followed by 30 min sonication. Afterward, 5 μL of the MWCNT solution was deposited on a GCE, which was subsequently dried in vacuo for 30 min. For DCNQ-based bioanode fabrication, 10 μL of a 10 mM DCNQ solution was deposited on the MWCNT-modified GCE and dried in vacuo for 30 min. Then, 15 μL of a mixture containing 50 mM TRIS/HCl pH 8.5, 2.75 mg/mL TeGDH, and 0.6 mg/mL dopamine, which was preincubated for 30 min at room temperature (RT), was deposited on the electrode. The modified GCEs were further incubated for 90 min at room temperature. For DCPIP-based bioanodes, 5 μL of a mixture containing 50 mM TRIS/HCl pH 8.5, 2.75 mM DCPIP, 2.75 mg/mL TeGDH, and 0.6 mg/mL dopamine, which was preincubated for 30 min at room temperature, was deposited on the electrodes. The modified electrodes were further incubated for 1 h at RT. For ABTS-mediated BOD biocathodes, a 5 μL mixture containing 50 mM TRIS/HCl pH 8.5, 0.8 mg/mL BOD, 120 μM ABTS, and 66 μg/mL dopamine was deposited on a GCE modified with MWCNTs (as described above), which was then incubated for 1 h at RT. For activity and saturation curve measurements, the DCPIP- or DCNQ-based bioanodes were incubated in 20 mL of KPi 0.1 M pH 7 for 5 min and then measured via cyclic voltammetry (CV) (from −0.3 to 0.2 V vs Ag/AgCl, 5 mV/s). The electrodes were measured without an analyte, as well as under increasing glucose concentration. CV measurements were performed a minute after glucose addition. DCNQ-based bioanodes were tested using chronoamperometry (CA) at 0 V vs Ag/AgCl. Prior, the electrodes were immersed in 0.1 M KPi pH 7 for 5 min. After current stabilization, the following analytes were added to the solution in sequence: 68 μg/mL uric acid, 2.5 mM glucose repeated four times, 0.1 mM ascorbic acid, 1.1 mM acetaminophen, and 5 mM glucose repeated twice. The solution was homogenized via pipetting during a brief pause in the measurement. GCE–DCNQ–TeGDH stability measurements were performed by CA (0 V vs Ag/AgCl) in 20 mL of 0.1 M KPi pH 7 for ∼24 h. After 14 h, 10 mM glucose was added, and the measurement continued for 10 additional hours.

### EBFC Measurement

A DCNQ-based bioanode and a biocathode were prepared as mentioned above and placed inside a triple-necked flask filled with 15 mL of KPi 0.1 M pH 7. The cell was measured via linear sweep voltammetry (LSV) (59 s hold, 2 mV/s, from 0 to 0.5 V vs open circuit voltage, (OCV).) using the biocathode as a reference. The measurement was performed under atmospheric conditions and O_2_ enrichment.

## Results and Discussion

**Figure 1:**
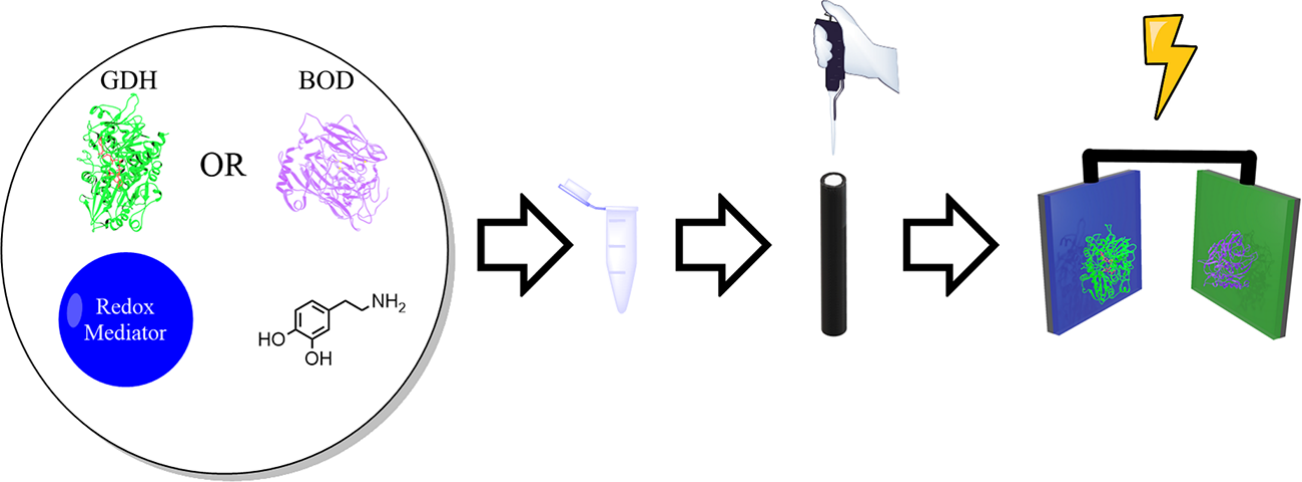
Fabrication of a polydopamine-based biosensor. TeGDH or BOD is mixed with their respective redox mediator and polydopamine and then deposited on the GCE-MWCNT surface. Both biosensors can be combined to form a biofuel cell.

**Figure 2:**
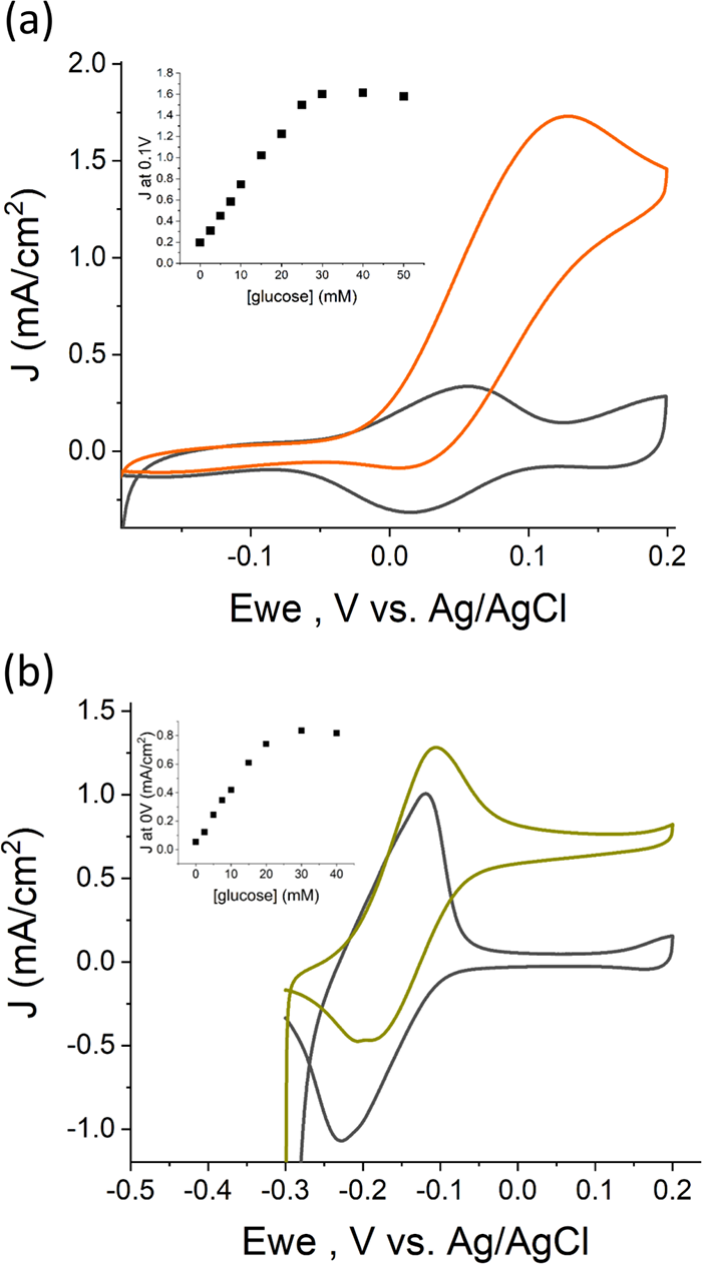
Cyclic voltammetry (CV) of a TeGDH-based biosensor. (a) CV measurement of a DCPIP-based sensor with (orange) and without (black) addition of 40 mM glucose. The inset represents a calibration curve at 0.1 V versus Ag/AgCl based on CV measurements with varying glucose levels. (b) CV measurement of a DCNQ-based sensor with (beige) and without (black) addition of 40 mM glucose. The inset represents a calibration curve at 0 V versus Ag/AgCl based on CV measurements with varying glucose levels.

The TeGDH sequence was cloned into pET29b, after which it was overexpressed in *E. coli* and purified using affinity and gel filtration columns (Figures S1–S3 present the colony PCR, absorbance spectra, and SDS-PAGE). The protein was then concentrated to 11 mg/mL and its activity was determined. *K*_M_ and *k*_cat_ were calculated to give values of 17.5 mM and 886 s^–1^, respectively. These results are in agreement with previous findings.^28^ By following the spectral absorbance of the protein and the FAD cofactor in the UV/vis range, we could estimate that 93.5% of the purified enzyme was active (Figure S2). Using the purified enzyme as a biocatalyst for glucose oxidation, we constructed an amperometric biosensing device, as depicted in Figure 1. For that, we first modified the glassy carbon electrodes (GCE) with multiwalled carbon nanotubes (MWCNTs). The MWCNTs increase the practical surface area by a factor of 14.3 (Figure S4). Then, a mixed solution of TeGDH, 2,6-dichlorophenolindophenol (DCPIP), and dopamine were preincubated for 30 min and then deposited on the GCE-MWCNT surface. The dried electrode was then tested for activity by following the bioelectrocatalytic currents in the absence or presence of glucose, as shown in Figure 2. By plotting the achieved bioelectrocatalytic current versus the increased glucose concentration, a linear correlation could be obtained in the range of 0–20 mM glucose, which is the desired detection range for a diabetic patient. In an earlier report, TeGDH was directly deposited on an electrode to give DET-type bioelectrocatalytic currents.^30^ By depositing the purified TeGDH on a MWCNT electrode, we could only measure low bioelectrocatalytic currents at a high overpotential of 0.4 V versus Ag/AgCl (Figure S5). While DCPIP enables the efficient MET process, lower potentials are advantageous for either sensing or BFC devices. To reach lower potentials, DCPIP was replaced with redox mediators consisting of more negative potential such as methylene blue, anthraquinone sulfonate, and DCNQ. While the potential of all tested redox molecules could thermodynamically mediate the electron transfer process between the flavin active site and the electrode, only DCNQ enabled efficient bioelectrocatalytic currents with an onset potential at ∼−0.2 V, as depicted in Figure 2b. The thionine molecule was also tested as a redox mediator. While the molecule has a structural similarity to methylene blue, it facilitated bioelectrocatalytic currents that are similar to DCNQ (see Figure S6).^19^ DCNQ has low solubility in an aqueous solution and has a redox potential that is 200 mV more negative than DCPIP. These are advantageous for the long-term stability of future constructed devices, and therefore, DCNQ was chosen as the redox mediator for the TeGDH-based bioanodes.

**Figure 3:**
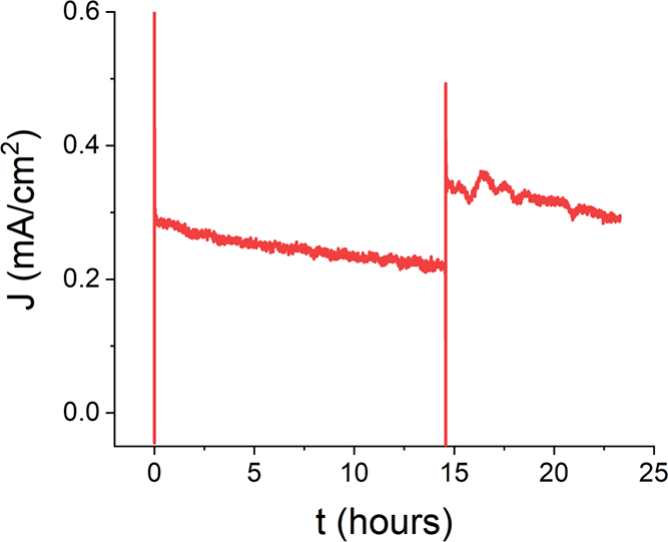
Chronoamperometry (CA) of a DCNQ-based glucose biosensor for 1 day in 10 mM glucose at 0 V versus Ag/AgCl. After 14 h, another dose of 10 mM glucose was mixed into the solution. The solution was homogenized via pipetting during a brief pause in the measurement.

**Figure 4:**
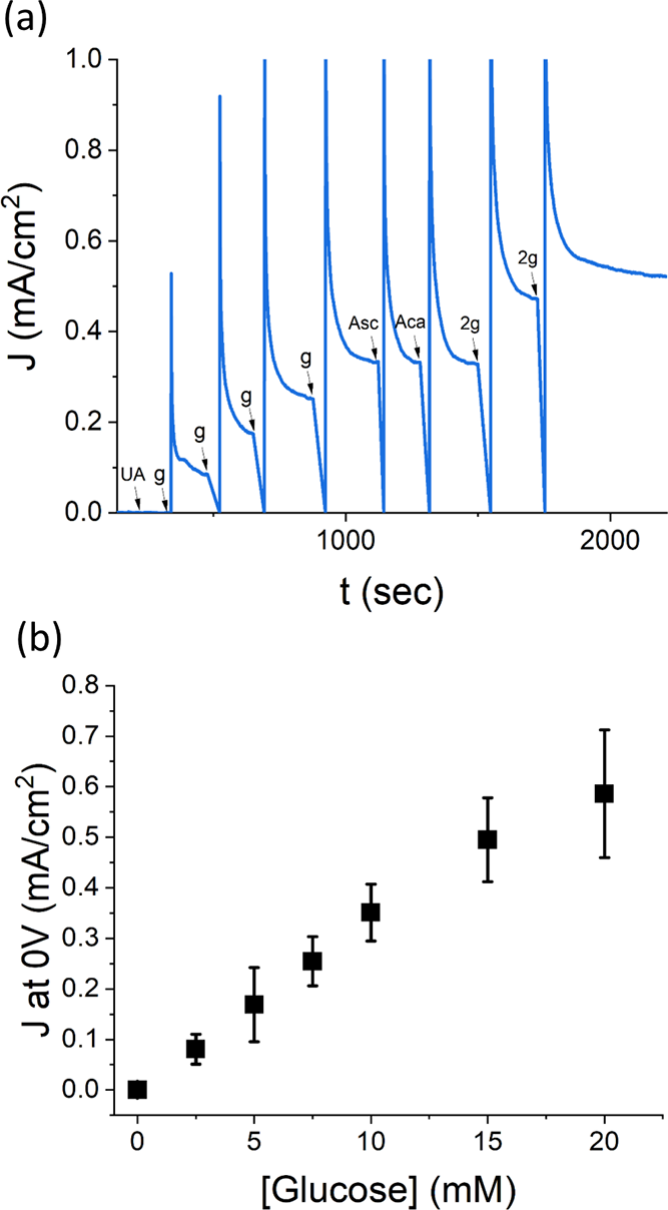
Interference assay for the DCNQ-based biosensor. (a) CA of the interference assay at 0 V versus Ag/AgCl. Analytes were added in the following order: UA—6.8 μg/mL uric acid, g—2.5 mM glucose, Asc—100 μM ascorbic acid, Aca—1.1 mM acetaminophen, and 2g—5 mM glucose. The solution was homogenized via pipetting during a brief pause in the measurement. (b) A plot of average current versus glucose concentration. The current values were taken from three separate CA measurements.

The DCNQ-based configuration was further characterized, and both redox mediator and enzyme content were analyzed. The DCNQ redox mediator amount was determined to be ∼0.78 nmol, while the protein content was 2 nmol (Figure S7). We then examined the stability of the designed MWCNTs/GDH/DCNQ biosensor. Chronoamperometry measurements at 0 V versus Ag/AgCl were applied while 10 mM glucose was present in the test solution. The generated bioelectrocatalytic current was stable with a systematic 22% drop for over 14 h, as shown in Figure 3. We further examined the bioanode’s long-term bioelectrocatalytic activity by the addition of a second glucose dose to the test solution. As depicted in Figure 3, a current jump coincides with the second addition of glucose, yet with lower intensity. Measurements of interference caused by biomarker molecules similar to the analyte are a key problem that needs to be addressed in any amperometric biosensor device. Therefore, we examined the TeGDH-based bioanode amperometric response while chemicals such as ascorbic acid, uric acid, and acetaminophen were present (Figure 4). The measurement revealed a small change in the biosensor’s sensing capability, and a slight deviation was found at 20 mM glucose, as shown in Figure 4b. This agrees with both our Michaelis–Menten curve (see Figure S8) and the literature. It should be noted that no bioelectrocatalytic currents were measured without the presence of the TeGDH enzyme using only MWCNTs, DCNQ, and polydopamine (Figure S9). While test strips are a common methodology used by patients to regulate their glucose concentration, continuous glucose monitoring devices are the present and the future of glucose regulation. These biosensing devices operate in the interstitial fluid (ISF) layer; therefore, we tested our designed configuration under a fluid that simulates the human ISF (Figure S10).^31^ As expected, lower bioelectrocatalytic currents were observed due to interactions with bovine serum albumin (BSA) proteins and high salts; nevertheless, the sensor was active and enabled glucose sensing without any additional coating step. Besides biosensing applications, the developed bioanode can be utilized in EBFC applications. The FAD-GDH enzyme has an advantage as compared to GOx-based devices due to its oxygen-independent catalytic activity. In recent years, several FAD-GDH-based BFCs were introduced. Oxygen insensitivity is a major advantage in biofuel cell devices, as it prevents the short-circuit reaction of oxygen in both bioanode and biocathode.^17,22,23^ For example, BFC devices were constructed using electropolymerization techniques, nanoporous gold, redox polymers, and osmium complexes bonded to polymeric chains.^32−38^ While major advances have been reached, for practical applications, a simple, low-cost construction methodology should be further realized.

**Figure 5:**
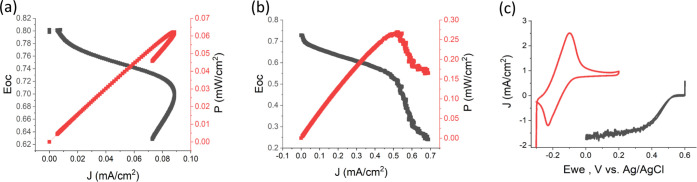
Power output of the TeGDH enzymatic biofuel cell. (a) Linear sweep voltammetry (LSV) describing the power output with 40 mM glucose under atmospheric conditions. (b) An LSV curve describing the power output with 40 mM glucose and enriched O_2_. (c) A CV curve of the TeGDH bioanode and the BOD biocathode under 40 mM glucose and enriched O_2_, respectively.

An EBFC was fabricated using bilirubin oxidase (BOD) from *M. verrucaria* as a biocatalyst for the biocathode. PDA was used as a scaffold polymeric layer to immobilize the BOD together with the ABTS redox mediator, as was recently shown.^39,40^ The TeGDH-based bioanode and the BOD-based biocathode were then conjugated through an external circuit to form an EBFC device. Using a polarization curve, we could measure the power output generated and further characterize it under both atmospheric conditions and O_2_ saturation. Compared to previously published work with TeGDH, the presented EBFC has reached higher power outputs, as shown in Figure 5. The maximal power reached 63 μW/cm^2^ under atmospheric conditions, which are 30 times higher than previously reported.^29^ By examining the cell under oxygen saturated conditions, a power output of 270 μW/cm^2^ was reached. The potential difference between the bioanode and the biocathode has reached 720 mV, which is dictated by the difference between DCNQ and ABTS redox potentials (Figure 5c).

The designed biosensor possessed a good linear response in the range between 0 and 20 mM. This range is mandatory for any future applications toward CGM devices.^41,42^ While the biosensor exhibits good stability for at least 24 h, we can still see current losses that should be considered in any future device development. The use of a programmed electronic device that can correct the current drop should solve the issue. By comparing the cloned TeGDH enzyme with other FAD-GDHs that are available for commercial applications (e.g., *A. Sp.* from Sekisui Diagnostics), we could achieve similar stability and bioelectrocatalytic activity.^43^ While these results are promising, genetic manipulation or directed evolution techniques might lead to improved results in terms of enzyme stability or turnover rate. By comparing the obtained TeGDH-based bioanode results with a TeGDH bioanode lacking redox mediators, we can conclude that in our configuration, only MET can establish efficient electrical communication with the enzyme. The strong π–π interactions of DCNQ, TeGDH, and polydopamine with MWCNTs allow improved stability, as shown recently.^44^ Moreover, the power output using MET was higher than DET due to a better ET process and lower overpotential. The PDA-based configuration yielded improved currents, thus showing its advantage over DET with the presented methodology. The bioanode remained stable for over 20 h of operation, and spiking the sensor with additional glucose amounts revealed that the sensor is still active and responsive. The effect of interferents was also examined and was found to be negligible. This was indeed expected, as the low voltage applied in the developed configuration should not lead to the oxidation of interferents. It should also be noted that a linear response to low glucose concentrations at the range of 1–5 mM was obtained (Figure S11). This could be an important range in cases of hypoglycemia. The biosensor was shown to function on Toray paper as well, whose low costs and porous structure make it attractive for applicative uses (Figure S12). While sensing an important biomarker like glucose is extremely important, a multisensor capable of simultaneous, varied biomarker sensing will have a bigger impact on human health. It may also provide a valid platform for physicians, providing fast point of care results with a wide scope that should allow better treatment.^45−48^ The developed EBFC has led to a maximal power output of 270 μW/cm^2^, which is 8 times higher than a previously reported system.^22^ By examining the obtained bioelectrocatalytic currents, we can conclude that the developed bioanode limits the cell performance and should therefore be improved.

## Conclusions

We have cloned, overexpressed, and purified the FAD-GDH from *T. emersonii* in *E. coli*. The enzyme was characterized biochemically and further incorporated in an amperometric biosensing device. The biosensor was tested and showed good stability for at least 20 h with a linear response to glucose in the range of 0–20 mM. We tested the enzyme bioelectrocatalytic activity under a variety of redox mediators and in a DET configuration. We concluded that under the tested conditions, DET yielded low bioelectrocatalytic currents with a much higher overpotential than in the MET configuration. We further showed the construction of an EBFC device that is based on a bilirubin oxidase biocathode coupled with a TeGDH bioanode. The maximal biofuel cell performance reached 270 μW/cm^2^ under an oxygen atmosphere.

## Supporting Information Available

Reagents and instrumentation (Section S1); TeGDH cloning and overexpression (Section S2); TeGDH purification, SDS-PAGE, absorbance and FAD-GDH ratio (Section S3); measurement of increased surface area after MWCNT addition (Section S4); direct electron transfer electrode preparation and measurement (Section S5); bioanode fabrication (Section S6); quantification of immobilized protein on the electrode (Section S7); Michaelis–Menten curve (Section S8); glucose oxidation capabilities of polydopamine (Section S9); interstitial fluid (ISF) measurements (Section S10); glucose sensing at low concentrations (Section S11); biocathode fabrication (Section S12); Toray paper bioanode fabrication (Section S13); bioanode saturation curve (Section S14); quantification of DCNQ deposited on the electrode (Section S15); DCNQ-based bioanode interference assay (Section S16); DCNQ-based bioanode stability assay (Section S17); enzymatic biofuel cell fabrication and measurement (Section S18); plasmid map and full enzyme sequence (Section S19) (PDF)
