# Supplementary material for: Utilization of FAD-Glucose Dehydrogenase from T. emersonii for Amperometric Biosensing and Biofuel Cell Devices
Source: Anal Chem. 2021 Aug 12;93(33):11585–91. doi: 10.1021/acs.analchem.1c02157 (PMC8631703; doi:10.1021/acs.analchem.1c02157)
Supplement: Supplementary file 1 — ac1c02157_si_001.pdf [file ac1c02157_si_001.pdf]

Supporting information - Utilization of FAD-Glucose Dehydrogenase from T. emersonii for Amperometric Biosensing and Biofuel Cell Devices

Roy Cohen[a], Rachel E. Bitton[a], Nidaa S. Herzallh[a], Yifat Cohen[a], Omer Yehezkeli[a,b,c]*

a. Faculty of Biotechnology and Food Engineering, Technion –Israel Institute of Technology, Haifa 3200003, Israel

b. Russell Berrie Nanotechnology Institute, Technion –Israel Institute of Technology, Haifa 3200003, Israel

c. The Nancy and Stephen Grand Technion Energy Program Israel Institute of Technology, Haifa 3200003, Israel

Table of Contents

Section 1. Reagents and instrumentation......S2

Section 2. TeGDH cloning and overexpression......S2

Section 3. TeGDH purification, SDS PAGE, absorbance and FAD-GDH ratio......S4

Section 4. Measurement of increased surface area after MWCNT addition......S6

Section 5. Direct electron transfer electrode preparation and measurement......S6

Section 6. Bioanode fabrication......S7

Section 7. Quantification of immobilized protein on the electrode......S8

Section 8. Michaelis-Menten curve......S9

Section 9. Glucose oxidation capabilities of poly-dopamine......S10

Section 10. Interstitial fluid (ISF) measurements......S10

Section 11. Glucose sensing at low concentrations......S11

Section 12. Biocathode fabrication......S11

Section 13. Toray paper bioanode fabrication......S11

Section 14. Bioanode saturation curve......S12

Section 15. Quantification of DCNQ deposited on the electrode......S12

Section 16. DCNQ-based bioanode interference assay......S13

Section 17. DCNQ-based bioanode stability assay......S13

Section 18. Enzymatic biofuel cell fabrication and measurement......S13

Section 19. Plasmid map and full enzyme sequence......S14

References......S15

Section 1. Reagents and instrumentation

Glassy carbon electrodes (GCE, 3 mm diameter) were purchased from CH-Instruments. Dichlorophenolindophenol (DCPIP), dopamine, Uric acid and D-Glucose were purchased from Sigma-Aldrich. Dimethylformamide (DMF) was purchased from Bio-lab. Multi walled carbon nanotubes (MWCNTs) were purchased from Nanointegris (MWCNTs, 99 wt%, <20 nm OD). 2,3-dichloro-naphthoquinone (DCNQ) 98% was purchased from Acros Organics. Thionine acetate was purchased from sigma. Acetaminophen was obtained via crushing a 500 mg commercial paracetamol tablet (Teva pharmaceuticals, Israel). All chemicals and reagents were used without further purification. One and 0.05 micron alumina beads were purchased from BAS (Japan) and CH-instruments (USA), respectively. Bilirubin Oxidase from Myrothecium verrucaria (BOD), and 2,2'-azino-bis(3-ethylbenzothiazoline-6-sulfonic acid) (ABTS) were purchased from SIGMA Life Science. Ni-NTA beads, SDS-page ready-made gels (4-20%) and sample buffer were purchased from Genscript. Coomassie brilliant blue was purchased from Fischer bioreagents. PM2500 Protein marker was purchased from SMOBio. Agarose was purchased from Lonza. SybrSafe DNA staining reagent and ascorbic acid were purchased from Thermo Fischer.

All graphs were prepared with Origin software (Originlab, USA). All electrochemical measurements were performed using the Biologic SP-200 potentiostat, supported by EC-lab software (BioLogic, France). Protein purification was performed using AKTA GO FPLC (Cytiva) equipped with Superdex 200 column (Cytiva). Simulations of TeGDH protein structure were performed using the SWISS-MODEL website[1–8], and estimation of the TeGDH extinction coefficient was performed using the ProtParam website[9]. Signal peptide identification and removal was performed using SignalP[10]. DNA sequences and primer design were performed using the benchling website. Codon optimization was performed using the IDT codon optimization tool.

Section 2. TeGDH cloning and overexpression

The Thermophilic *Talaromyces emersonii* Glucose Dehydrogenase (TeGDH) gene was externally synthesized and cloned into pET29b (Twist Bioscience) using the sequence from entry LC069047 (DDBJ – DNA Database Bank of Japan) after codon optimization and

removal of the signal peptide (AA 2-17). Competent *E. coli* BL-21 (DE3) cells were transformed with pET29TeGDH and negatively selected using kanamycin agar plates. Surviving colonies were then positively selected via colony PCR, using the following primers: Forward – **5'** TTA TGC GAC TCC TGC ATT AG **3'**, Reverse – **5'** GTG CCA TAT GTA TAT CTC CTT C **3'**. The forward primer starts from the pET29 backbone to verify orientation as well as successful cloning.

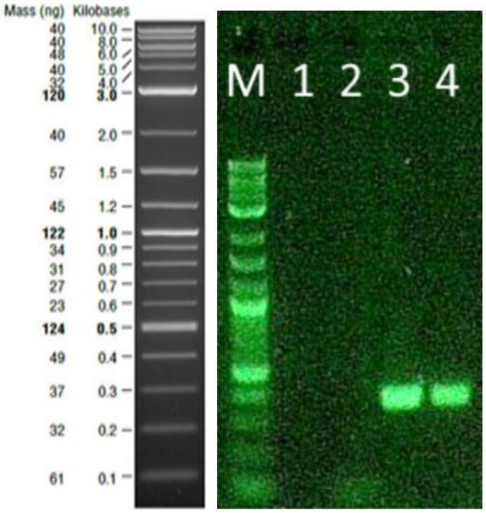

Figure S1 Colony PCR results. To the right, an agarose gel (1%), where M is the DNA marker lane and each other lane represents a single clone. Successful transformation is indicated by a strong band around 400bp. To the left is the legend for the DNA marker.

For TeGDH overexpression, a starter of 50 ml of LB was first seeded with colony 4 (see Figure S1 above), inoculated with 50 µg/ml Kanamycin and then incubated at 37°C, 180 RPM, overnight. The entire starter was mixed into a 1L Erlenmeyer flask containing 500ml Terrific Broth (without glycerol), which was inoculated with 50 µg/ml kanamycin, 1 mM MgCl, 1 mM CaCl and 10mM glucose. The cells were further incubated at 37°C, 180 RPM for approximately 7 hours. Then, the cells were induced with 500 µl 0.3M IPTG for 18-20 hours of incubation while stirred at RT to maintain aerobic conditions.

Then, 50 ml of cells were centrifuged using four 50 ml tubes (5000 RPM, 6 min, 4°C), and the supernatant was removed. The cell pellet was slightly yellowish due to FAD production. Then additional 50ml of cell suspension was added into each tube followed by a centrifugation step and supernatant removal. The process was repeated until no cell

suspension remained. All four tubes were frozen at 80°C after supernatant removal until further use.

Section 3. TeGDH purification, SDS PAGE, absorbance and FAD-GDH ratio

TeGDH cells from a frozen tube were resuspended in 20ml lysis buffer (50 mM Kpi pH 7.5, 10 mM Imidazole, 300 mM NaCl) and disrupted by ultrasonication (30% amplitude, 15 sec on 30 sec off, 11 min total on). Removal of cell debris was performed by centrifugation (10,000 g, 30 min, 4°C), after which the supernatant was kept.

The supernatant was passed through a Ni-NTA column, then washed with 7ml cold solution of 20 mM Kpi pH 7.5 containing 25 mM Imidazole and 300 mM NaCl. Aliquots of a cold 200 μl solution consisting of 20 mM Kpi (7.5 pH), 300 mM imidazole, 300 mM NaCl were used to elute the protein. This resulted in two yellow-colored main fractions.

The two main fractions were combined and further purified using a Superdex 200 column, equilibrated with 20mM KPi pH 7.5 containing 150mM NaCl. Using an isocratic gradient at 0.5ml/min, the protein was eluted after ~16ml (see below).

The main fractions of TeGDH were combined and concentrated using the PALL centricon (4000 RPM, 4°C, 30 min, 10kDa cutoff). The final concentration was found to be ~11mg/ml.

The purified protein was diluted 50 times and measured via spectroscopy. The typical absorbance of TeGDH was estimated at 1.636 per 1 mg protein/ml. The concentration of TeGDH at 280 nm ($\varepsilon280=103.7\ mM^{-1}\ cm^{-1}$) and that of FAD at 460 nm ($\varepsilon460=10\ mM^{-1}\ cm^{-1}$)[11] was calculated. [FAD] was divided by [TeGDH] to yield the cofactor-protein ratio.

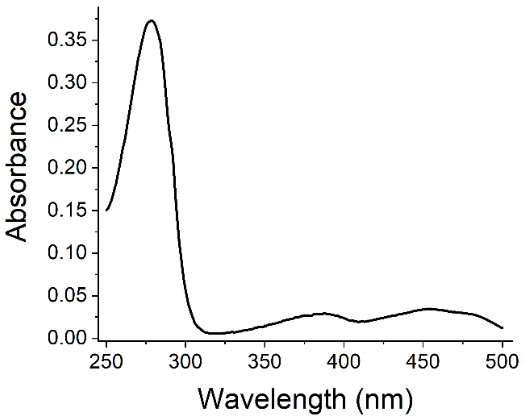

Figure S2. Purified TeGDH absorbance spectrum after dilution.

After purification, 10µl of protein were mixed with 10µl of sample buffer and 20µl DDW. The mixture was heated for 10 min at 70°C, after which 25µl were loaded onto the SDS-PAGE gel. The gel was subjected to 120V for 90 min, then immersed into DDW and heated using a commercial microwave for 30 sec. The gel was then gently stirred in a container for 5 min, after which the DDW was removed. This process was repeated 3 times total. The gel was then immersed in a Coomassie staining solution, heated with a microwave for 20 sec, and gently stirred for 30min. The gel was washed several times with DDW, then incubated with DDW overnight to wash off the excess stain.

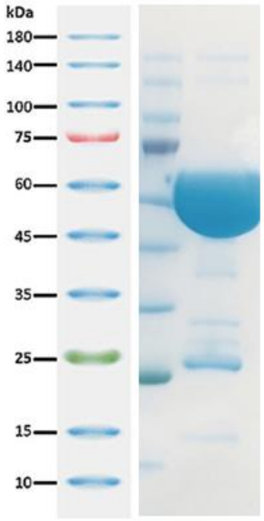

Figure S3. SDS-PAGE of TeGDH. The left lane represents the protein marker (see legend to the left). The right lane represents TeGDH after Ni-NTA and Superdex 200 purification.

Section 4. Measurement of increased surface area after MWCNT addition

A GCE was measured via CV (20mL ISF, from -0.3 to 0.2V vs. Ag/AgCl, 5 mV/s), after which 5µl of 5mg/ml MWCNT were deposited on it. The GCE was then dried *in vacuo* for 30 min. The same GCE was measured again using the same conditions to estimate the increase in surface area, which relates to capacitance in a linear fashion.

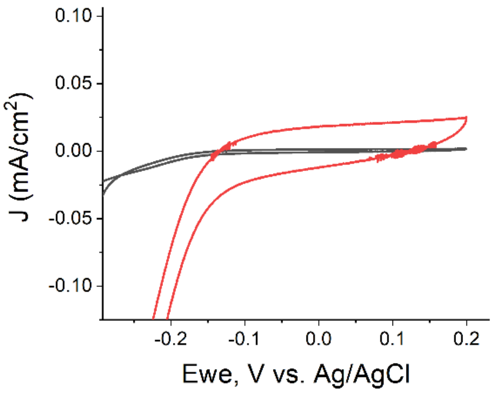

Figure S4. CV Measurement of the GCE before (black) and after (red) addition of MWCNT.

Section 5. Direct electron transfer electrode preparation and measurement

After preparation of GCE modified with MWCNTs as described in section five, 5 µl of TeGDH solution (11mg/ml) were deposited on the electrode. The electrode was incubated for 30min under atmospheric conditions, then measured via CV (in 20mL KPi 0.1 M pH 7.5, from -0.3 to 0.4V vs. Ag/AgCl, scan rates were 5 and 50 mV/s) before and after addition of 40mM glucose. The CV curves for the 5mV/s scan rate were smoothed twice using Origin's adjacent averaging to remove background noise.

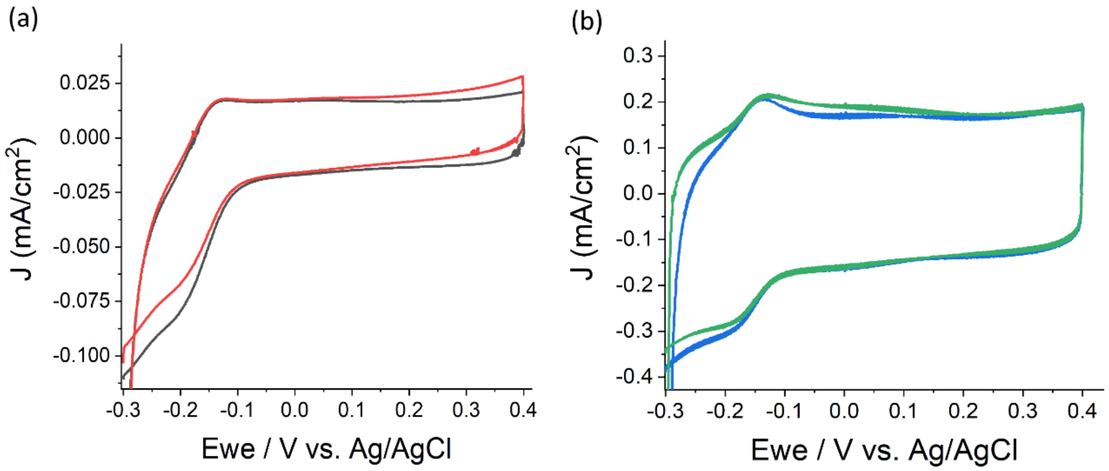

Figure S5. Cyclic voltammetry measurements of TeGDH DET electrodes, scanned at different scan rates. (a) CV at 5mV/s before (black) and after (red) addition of 40mM glucose. (b) CV at 50 mV/s before (blue) and after (green) addition of 40mM glucose

Section 6. Bioanode fabrication

GCEs were polished with 1 µm and 0.05 µm alumina beads in sequence. MWCNTs suspension (5 mg/ml) was prepared by dissolving MWCNTs in DMF, followed by sonication for 30 min. Afterwards, five µl of the MWCNTs solution were deposited on each GCE, which were then dried *in vacuo* for 30 min.

For DCNQ-based bioanode fabrication, 10 µl of 10 mM DCNQ solution was deposited on the MWCNTs modified GCE and dried *in vacuo* for 30 min. Then, 15 µl of a mixture containing 50 mM TRIS/HCl pH 8.5, 2.75 mg/ml TeGDH, and 0.6 mg/ml dopamine, which was pre-incubated for 30 min at room temperature, was deposited on the electrode. The modified GCEs were further incubated for 1.5 hours at room temperature.

For DCPIP-based bioanodes, 5µl of a mixture containing 50 mM TRIS/HCl pH 8.5, 2.75 mM DCPIP, 2.75 mg/ml TeGDH and 0.6 mg/ml dopamine, which was pre-incubated for 30 min at room temperature, was deposited on the electrodes. The modified electrodes were further incubated for one hour at RT.

Thionine-based bioanodes were prepared exactly like DCPIP based bioanodes, except for using Thionine instead of DCPIP at the same concentration.

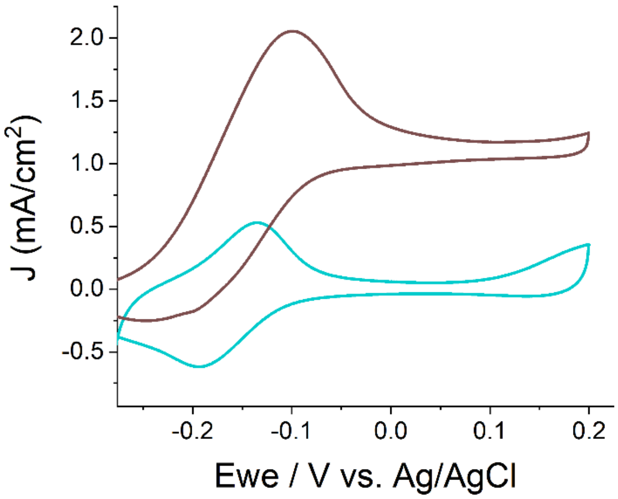

Figure S6. CV of a Thionine-based bioanode before (teal) and after (brown) addition of 40 mM glucose.

Section 7. Quantification of immobilized protein on the electrode

As most of the bioanode components have strong aqueous absorbance at 280nm (DCNQ is slightly soluble in water due to hydrogen bonds), protein adsorption was quantified using an indirect method. Two DCNQ-based bioanodes were prepared, where one mix solution contained DDW instead of enzyme. Both electrodes were soaked in two separate Eppendorf tubes containing 200 µl KPi 0.1 M pH 7.5 for 1h. Both electrodes were removed from the tubes, after which both solution were measured via spectroscopy. The spectrum of the non-GDH containing solution was subtracted from the other to receive the absorbance of the desorbed protein. The total amount of desorbed TeGDH was calculated at 280nm using the ext' coeff' mentioned above.

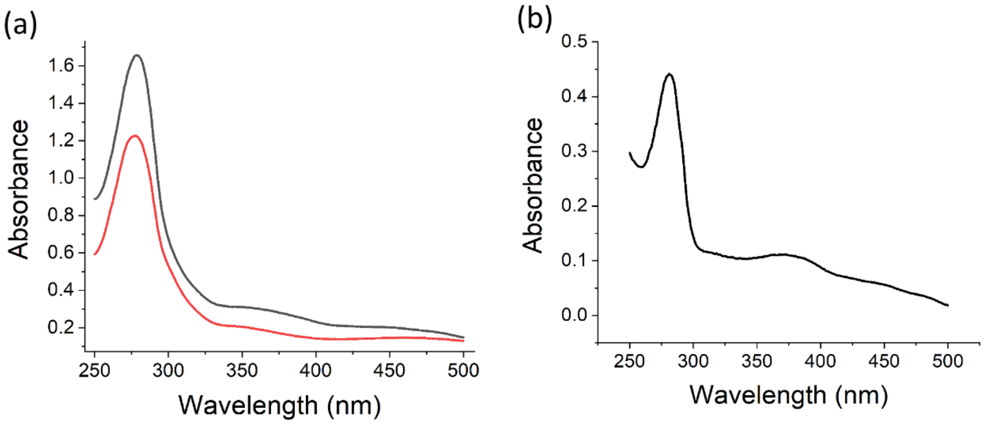

Figure S7. Indirect quantification of immobilized TeGDH. (a) Spectrum of the desorbed elements with (black) and without (red) TeGDH in the mix. (b) The subtraction of both spectra, which allows for an approximation of desorbed TeGDH.

Section 8. Michaelis-Menten curve

In order to determine kinetic enzymatic activity, absorption at 600 nm was measured in a stock solution (0.3 mM DCPIP, 100 mM Kpi, 10 µg/ml TeGDH) that contained rising glucose concentrations, (10, 100, 200 and 500 µM, as well as 1, 2, 5, 10, 20 and 40 mM). DCPIP concentration was doubled at higher glucose concentrations (60 and 80 mM). A Michaelis-Menten curve was established by plotting reaction rate (decreased OD_{600} signifies [DCPIP] reduction, and is quantified using ε_{600}=20.6 mM^{-1} cm^{-1})[12] vs. [glucose], as can be seen in Figure S4. K_M was determined using the Michaelis Menten curve.

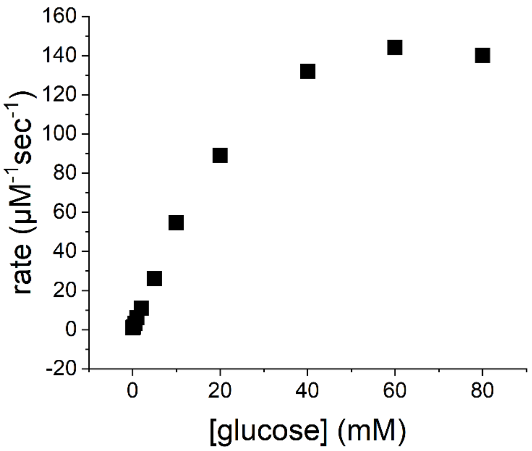

Figure S8. TeGDH Michaelis-Menten curve.

Section 9. Glucose oxidation capabilities of poly-dopamine

A bioanode was prepared as mentioned above, except DDW was added instead of TeGDH at equal volume. The bioanode was examined via CV (20mL ISF, from -0.3 to 0.2V vs. Ag/AgCl, 5 mV/s) before and after addition of 40mM glucose.

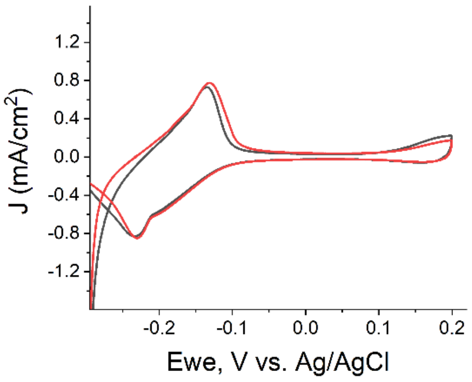

Figure S9. CV Measurement of the bioanode before (black) and after (red) addition of 40mM Glucose.

Section 10. Interstitial fluid (ISF) measurements

An ISF-like solution was freshly prepared according to Bennet and Leech[13]. A DCNQ-based bioanode was measured via CV (20mL ISF, from -0.3 to 0.2V vs. Ag/AgCl, 5 mV/s) before and after addition of 40mM glucose, as seen in Figure S9.

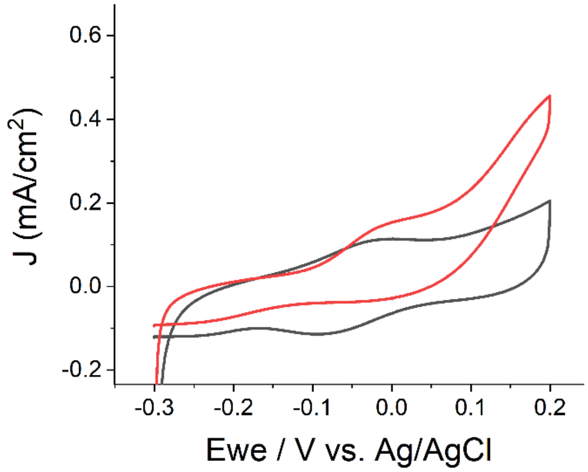

Figure S10. Cyclic voltammetry measurements of DCNQ based bioanode in ISF before (black) and after (red) addition of 40mM glucose.

Section 11. Glucose sensing at low concentrations

A bioanode was prepared as mentioned above. The bioanode was measured using CA at 0V, where increments of 1mM glucose were added in sequence. The solution was homogenized between each addition via pipetting.

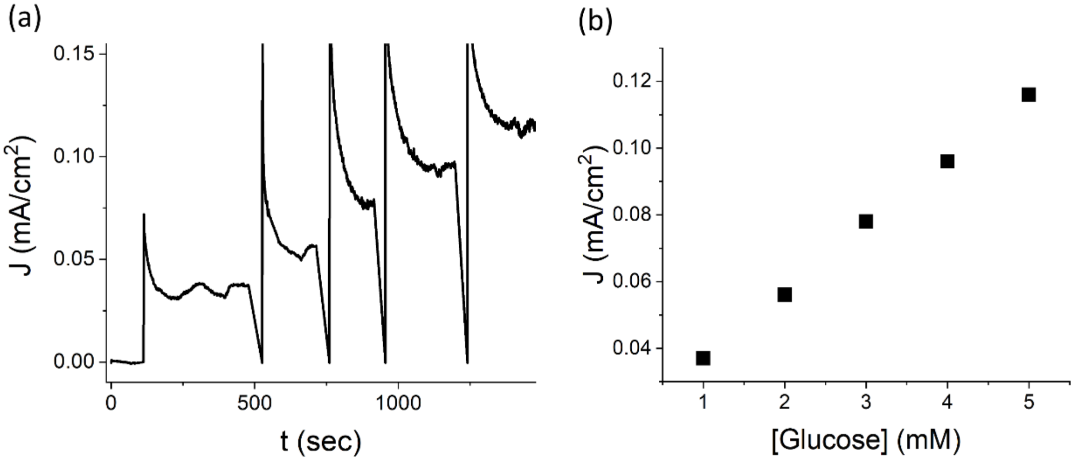

Figure S11. CA Measurement of the bioanode in low concentrations of glucose.

Section 12. Biocathode fabrication

For ABTS mediated BOD biocathodes, a 5µl mixture containing 50 mM TRIS/HCl pH 8.5, 0.8 mg/ml BOD, 120 µM ABTS, and 66 µg/ml dopamine was deposited on GCE modified with MWCNTs (as described above), which was then incubated for one hour at RT.

Section 13. Toray paper bioanode fabrication

Toray paper was cut to $0.5cm^2$ strips (0.5cm*1cm) and immersed in 10mM DCNQ (dissolved in acetone). The strips were immersed inside a 1.5ml Eppendorf tube. The strips were then left to dry for 3h under atmospheric conditions. Then, 30µl of the mix used for DCNQ-based electrode fabrication (see section 5) were deposited on one end of the strip. The strip was left to dry for two more hours, then measured via CV (20ml KPi 0.1 M pH 7, scanning from -0.3V to 0.2V vs. Ag/AgCl, 5mV/s) before and after addition of 40mM glucose.

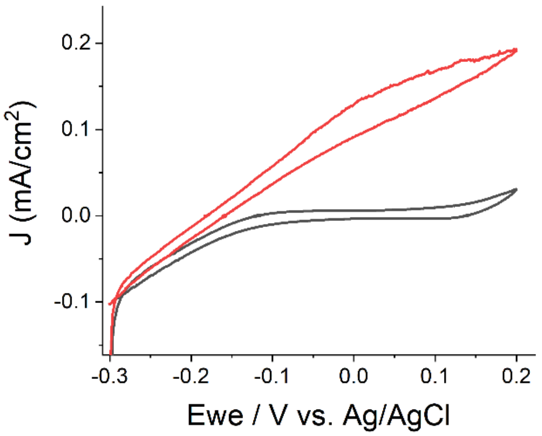

Figure S12. Cyclic voltammetry measurement curves of Toray paper DCNQ-based bioanode before (black) and after (red) addition of 40 mM glucose to the cell solution.

Section 14. Bioanode saturation curve

For TeGDH activity measurements, the DCPIP or DCNQ based bioanodes were incubated in 20ml Kpi 0.1 M pH 7 for five minutes and scanned measured via CV (from -0.3V to 0.2V vs. Ag/AgCl, 5mV/s). The electrodes were measured before and after the addition increasing glucose concentration. The electrodes were incubated for 1 minute after each glucose addition before CV measurement.

Section 15. Quantification of DCNQ deposited on the electrode

The amount DCNQ adsorbed on the electrode (in moles) was quantified according to the following equation:

$$(1)\ n_{mediator} = Q/z \cdot F$$

Where Q is the overall charge involved in the oxidation, z is the number of electrons involved in each reaction (2 in our case) and F is the Faraday constant. Q was derived from the integrated current involved in the oxidation of the mediator, which can be detected via CV. The total charge was calculated as such:

$$(2)\ n_{DCNQ} = \frac{Q}{z \cdot F}$$

$$(3)\ dQ = I \cdot dt$$

$$(4)\ \nu = V/t$$

(5) $Q = \int dI \cdot t = \int \frac{dI \cdot dV}{v}$

Where t is time, V is voltage and v is the scan rate.

Section 16. DCNQ-based bioanode interference assay

DCNQ mediated bioanodes were subjected to CA (0V applied voltage) after five min immersion in 0.1 M Kpi pH 7. After current stabilization, the following analytes were introduced into the solution in sequence: 68 µg/ml Uric Acid, 2.5 mM glucose repeated four times, 0.1 mM Ascorbic acid, 1.1 mM Acetaminophen and 5 mM glucose repeated twice. The measurement was briefly paused before each analyte addition to allow homogenization of the solution via pipetting.

Section 17. DCNQ-based bioanode stability assay

GCE-DCNQ-TeGDH stabilization measurements were scanned via CA (0V vs Ag/AgCl) in 20 ml 0.1 M Kpi pH 7 for 20 hours. After 14 hours, 10 mM glucose was added and the measurement continued for 10 additional hours.

Section 18. Enzymatic biofuel cell fabrication and measurement

A DCNQ-based bioanode and a biocathode were prepared as mentioned above and placed inside a triple-necked flask filled with 15ml KPi 0.1 M pH 7. The cell was measured via linear sweep voltammetry (59 sec hold, 2mV/s, from 0 to 0.5V vs. EOC) using the biocathode as a reference. The measurement was taken under atmospheric conditions and under O_2 enrichment.

Section 19. Plasmid map and full DNA sequence

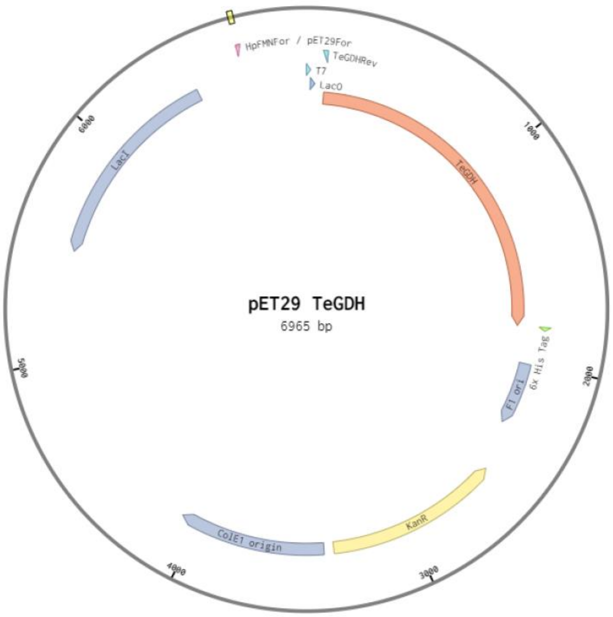

Figure S13. Schematic description of the pET29TeGDH plasmid structure.

The TeGDH coding sequence is:

ATGGCACCGTTGTGCCAAACAGCGAGTTCGTCGTATGACTATATTGTGGTTGGCGGGGGCACTAGTGGTTTGGTTGTTGCGAACCGTCTGAGTGAGAATCCGAAC
GTCTCTGTACTGGTAATCGAGGCCGGCGATTCCGTTTATAACAACGCGAATGTCACCAATGTCAACGGTTATGGCCTTGCGTTTGGCACGCCGATTGATTGGCAAT
ATCAAAGCACGAACCAAACATACGCCGGTAATACCCGCCAAACCCTGCGTGCAGGCAAAGCGTTGGGTGGAACATCTACCATTAATGGTATGGCGTACACCCGT
GCACAAGATGTTCAGATTGATGCGTGGGCGGCGATTGGGAACGATGGATGGGATTGGAGCTCTCTGTGGCCGTATTACCTTAAATCGGAAGCATTTACGGCTCCG
AATCAGACCCAGCGTGCGGCGGGAGCCAGCTATAATCCGGCTTATCACGGTGTGACTGGCCCTCTGCATGTTGGTTTCATTGAAATGCAGCCAAACAACTTGAGC
TCGATTTTGAACCAGACCTATCAGGCCCTGGGAGTACCGTGGACAGAAGATGTGAATGGAGGTAAGATGCGTGGCTACAATTTCTTCCCGAGCACCGTTGATGAC
GCCGCCGACGTGCGCGAAGATGCTGCACGTGCCTATTACTATCCATTTGAATCCCGCCCTAATCTTCGTGTAATGCTGAATACCCTGGCGAACCGCATTGTCTGGA
AGAACGAAACAAGCGGAGGCAACGTCACCGCGGATGGCGTAGAGGTTACTCCTCTGAATGGCACCGTGTGTCGCATTCAGGCGAACAACGAAGTTATTTTAAGC
GCGGGATCCCTGCGCTCACCAGGTATTTTGGAGCTTAGCGGTGTAGGCAACCCGTCCATTCTGAATAAATATAATATTCCAGTTAAAGTTAACCTGCCGACAGTG
GGAGAGAACATGCAGGATCAGATGTATAATGATGCGAGTGCAGAAGGCTACTCTGCAATTGCCGGCACTAAAAGTGTCGCCTATCCGTCGGTCACGGATTTATTT
GGTAATCGCACTTCGGCCGTGGCTGCTTCCGTGCAGAACCAACTGGCGCAGTACGCCGAAGCGGCGGCCAACCAATCACAGGGGACCATGAAGGCGTCTGATTT
GCAGCGCTTGTTTCAGATTCAGTATGATCTGATTTTCAAACAAGAGGTCCCGATTGCAGAGATTATTACTTACCCCACTGGTAATACATTAGCGGCTGGCTATTGG
GGCTTGTTACCGTTCGCGCGCGGATCGGTGCATATCGCGAGCGCCGATCCCACAGTCCAGCCGGTGATTAATCCAAACTACTTCATGTTCGATTGGGATGTTCAAC
AGCAGATCGGTACTGCCAAATTCATTCGCAACTTATACAAAACCGCCCCTCTGTCGTCCCTGGTAAAGAACGAGACCGAGCCGGGTTCGGCGGTACCAGAAGGC
GCATCAGACTCGGTTTGGGAGGCCTGGCTTAAAGAAACCTATCGCAGCAACTTTCACCCGGTGGGCACGGCGGCAATTATGCCGCGCTCGATCGGCGGTGTCGTT
GACGAGCGCTTACGTGTGTATGGCACTGCGAACGTGCGTGTGGTGGATGCCTCTGTTCTGCCGTTCCAGATTTGCGGCCACCTGACGAGCACGCTGTACGCCGTCG
CGGAACGCGCTTCAGATTTCCTGAAAGAAGATGCAGCCCGGTTGGGAAATCTCGAGCACCACCACCACCACCACTGA

References

(1) Bertoni, M. Modeling Protein Quaternary Structure of Homo- and Hetero-Oligomers beyond Binary Interactions by Homology. *Sci. Rep.* 15.

(2) Bienert, S.; Waterhouse, A.; Studer, G.; Bordoli, L.; Schwede, T. The SWISS-MODEL Repository—New Features and Functionality. 7.

(3) Mariani, V.; Biasini, M.; Barbato, A.; Schwede, T. LDDT: A Local Superposition-Free Score for Comparing Protein Structures and Models Using Distance Difference Tests. 7.

(4) Studer, G.; Rempfer, C.; Waterhouse, A. M.; Gumienny, R.; Haas, J.; Schwede, T. QMEANDisCo—Distance Constraints Applied on Model Quality Estimation. 7.

(5) Studer, G.; Tauriello, G.; Bienert, S.; Biasini, M.; Johner, N.; Schwede, T. ProMod3—A Versatile Homology Modelling Toolbox. *PLOS Comput. Biol.* 18.

(6) Waterhouse, A.; Bertoni, M.; Bienert, S.; Studer, G.; Tauriello, G.; Gumienny, R.; Heer, F. T.; Rempfer, C.; Bordoli, L.; Lepore, R.; Schwede, T. SWISS-MODEL: Homology Modelling of Protein Structures and Complexes. 8.
(7) Benkert, P.; Biasini, M.; Schwede, T. Toward the Estimation of the Absolute Quality of Individual Protein Structure Models. 8.
(8) Guex, N.; Peitsch, M. C.; Schwede, T. Automated Comparative Protein Structure Modeling with SWISS-MODEL and Swiss-PdbViewer: A Historical Perspective. *ELECTROPHORESIS* **2009**, *30* (S1), S162–S173. https://doi.org/10.1002/elps.200900140.
(9) Gasteiger, E.; Hoogland, C.; Gattiker, A.; Duvaud, S.; Wilkins, M. R.; Appel, R. D.; Bairoch, A. Protein Identification and Analysis Tools on the ExPASy Server. 38.
(10) Armenteros, J. J. A. SignalP 5.0 Improves Signal Peptide Predictions Using Deep Neural Networks. *Nat. Biotechnol.* **2019**, *37*, 8.
(11) Lee, J.-E.; Hofhaus, G.; Lisowsky, T. Erv1p from Saccharomyces Cerevisiae Is a FAD-Linked Sulfhydryl Oxidase. *FEBS Lett.* **2000**, *477* (1–2), 62–66. https://doi.org/10.1016/S0014-5793(00)01767-1.
(12) J.McD. Armstrong. The Molar Extinction Coefficient of 2,6-Dichlorophenol Indophenol. *Biochim Biophys Acta* **1964**, *86* (1), 194–197.
(13) Bennett, R.; Leech, D. Improved Operational Stability of Mediated Glucose Enzyme Electrodes for Operation in Human Physiological Solutions. *Bioelectrochemistry* **2020**, *133*, 107460. https://doi.org/10.1016/j.bioelechem.2020.107460.
